# Action and Non-Action Oriented Body Representations: Insight from Behavioural and Grey Matter Modifications in Individuals with Lower Limb Amputation

**DOI:** 10.1155/2018/1529730

**Published:** 2018-10-18

**Authors:** Liana Palermo, Antonella Di Vita, Maddalena Boccia, Federico Nemmi, Stefano Brunelli, Marco Traballesi, Roberto De Giorgi, Gaspare Galati, Cecilia Guariglia

**Affiliations:** ^1^Department of Medical and Surgical Sciences, Magna Graecia University of Catanzaro, Catanzaro, Italy; ^2^IRCCS Fondazione Santa Lucia, Rome, Italy; ^3^PhD Program in Behavioural Neuroscience, Department of Psychology, “Sapienza” University of Rome, Rome, Italy; ^4^Department of Psychology, “Sapienza” University of Rome, Rome, Italy; ^5^UMR1214–ToNIC Toulouse NeuroImaging Center, Université de Toulouse, Inserm, UPS, France

## Abstract

**Objective:**

Following current model of body representations, we aimed to systematically investigate the association between brain modifications, in terms of grey matter loss, and body representation deficits, in terms of alterations of the body schema (BS) and of non-action oriented body representations (NA), in individuals with lower limb amputation (LLA).

**Method:**

BS and NA (both semantic and visuospatial NA) were evaluated in 11 healthy controls and in 14 LLA, considering the impact of clinical variables such as prosthesis use. The association between BS and NA deficits and grey matter loss was also explored in LLA by using Voxel Based Morphometry analysis.

**Results:**

LLA's performance was fine in terms of semantic NA, while it showed behavioural impairments both in BS and visuospatial NA as compared to healthy controls. Interestingly the visuospatial NA performance was related to the amount of prosthesis use. NA deficits in terms of visuospatial body map processing were associated with grey matter reduction in left (lobule VIII) and right (crus II) cerebellum, while BS deficits were associated with grey matter reduction in right anterior cingulate cortex and the bilateral cuneus. No significant association was detected for semantic NA.

**Conclusion:**

The study of BS and NA representations after limb loss has informed our understanding of the different dynamics (i.e., adjustments to body change) of such representations, supporting current cognitive models of body representation. The clinical relevance of present findings is also discussed.

## 1. Introduction

Body representations are neural representations of the body formed within the human brain which arise from the integration and cross-reference of several inputs of different types, from sensorimotor to visual, from proprioceptive to interoceptive. This is a complex and unique process, central to our sense of self. How body is represented in the brain has long fascinated researchers but, notwithstanding the progress in understanding disorders and neural substrates, many aspects related to the development and dynamics of body representation are still a matter of debate. This complexity is reflected in the lack of a universally accepted taxonomy of body representations [1, 2]. However, a distinction between a body representation supporting action, also known as body schema, and a non-action oriented body representation is almost uncontroversial and widely accepted in the literature [3]. Converging scientific evidence from the neuropsychological literature on individuals with brain damage as well as behavioural and fMRI data on healthy participants and developmental studies supports the clinical and functional relevance of this distinction [1–12], being the body schema a mental representation relevant for action and interaction with the environment, and the non-action oriented body representations relevant for perception/recognition, sense of body ownership, and self-consciousness. Specifically, the body schema can be defined as a dynamic representation of body derived from multiple motor and sensory inputs that interact with the motor system in the genesis of actions [11]. The non-action body representations include, instead, representations such as the visuospatial body map, that is, a topographic representation of the body derived from visual information including body part boundaries and proximity relationships [11] and the body semantic representation that contains a lexical–semantic representation of the body including body part names, functions, and relations with artifacts [11]. These different types of body representation not only are based on different source of information but also seem to be supported by different neural networks. In particular a recent study has provided an up-to-date meta-analysis of 59 fMRI experiments on the neural substrates of body representations [3]. The authors found that overall the ability to mentally represent one's own body involves a wide network of areas in occipital, parietal, frontal, and temporal lobes and also that the body representation supporting actions (body schema, BS) selectively activates the primary motor area and the right extrastriate body area, while the non-action oriented body representation (NA) selectively activates the somatosensory primary cortex and the supramarginal gyrus.

In sum, even if different taxonomies and models have been proposed [2, 13], the distinction between BS and NA is straightforward for cognitive investigations of body representation. Indeed, it has been proposed that “it is highly plausible from an evolutionary perspective that body information processing evolved first to be used for action, and if one wants to study body representations, one should start with those that are action-oriented” [2]. In this light, the distinction between BS and NA offers a good frame to understand cognitive deficits following brain damage or body modifications (e.g., amputation). Indeed, both BS and NA can be affected by brain damage [4–6, 10, 11], but also by body changes, such as the loss of a limb. Clinically in brain damaged patients a BS deficit can result in difficulties in planning and performing motor actions, in adjusting posture and guiding movements, and in production, recognition, and imitation of gestures (e.g., ideomotor apraxia) while a NA deficit can result in difficulties in pointing to the body parts (e.g., autotopagnosia) and, more generally, in difficulties in actions on the body that are guided by vision and in daily activities that require the visual recognition of one's own body [1, 11]. Similar difficulties could be present in amputees, but studies evaluating the clinical manifestations of BS and NA deficits in amputees are lacking. However experimental studies with tasks probing BS (e.g., mental rotation of body parts [14–16]) and NA suggest the presence of BS and NA difficulties in amputees. Indeed, in tasks involving the mental rotation of body parts, individuals with upper limb amputation are slower and less accurate in making left/right* hand* judgments [15, 16], and individuals with lower limb amputation do not show the so-called laterality effect in making left/right* feet* judgments [14]. Since the mental rotation of body parts is a task that can be solved by imagining performing the actual movement [17]and that, as the actual movement, involves the body schema [11, 17], these previous studies suggest the presence of a BS deficit in amputees. Individuals with right lower limb amputation also show NA deficits in terms of difficulties in representing the positions and the relations among different body parts (visuospatial body map or structural body representation). Indeed, in the “Frontal Body-Evocation” subtest of the Body Representation Test [18] (for details see the Method section) these individuals fail to exactly locate different body parts (legs, hands, and so on) on a board where the head of a child is depicted. Notably, such a deficit is comparable to that showed by patients with brain damage due to stroke [10].

Overall, the above reviewed behavioural studies point to the idea that the loss of a limb can result in BS and NA deficits. This is possibly due to brain reorganization in specific body-related brain areas. Indeed, functional reorganization of the primary somatosensory cortex, with an expansion of adjacent cortical representational areas, is well documented in individuals with amputation [19, 20]. In addition, some studies also showed structural reorganization in grey matter following limb amputation (for an overview of studies see [21]). For example, Draganski and colleagues [22] found a decrease of thalamic grey matter in a sample of individuals with lower or upper limb amputations. Instead, in individuals with right upper limb amputation, Preißler and colleagues [23] showed a significant decrease of grey matter volume in the left primary motor cortex and in the right dorsolateral prefrontal cortex but also increased grey matter volume in different parts of ventral and dorsal pathways of the visual stream (e.g., left temporal pole, left fusiform cortex, right middle temporal cortex, and the right superior parietal cortex). Following lower limb amputation, decreased cortical thickness in V5/MT+ visual areas was showed by Jiang and colleagues [24], while decreased grey matter volume in cerebellum has recently been described by Di Vita and colleagues [21].

Although current studies allow us inferring that limb amputation may result in body representation deficits related to brain reorganization in specific areas, up until now no study has systematically investigated the association between brain modifications, in terms of grey matter loss, and body representation deficits, in terms of BS and NA alterations. In addition, although previous studies suggest that limb amputation can affect both BS [14, 16] and NA [10], no study has systematically analysed both BS and NA within the same sample. Specifically, it remains to be clarified whether amputation results in* a global body representation deficit* involving all of the body representations, linked to grey matter loss in the areas generally involved in body representation (e.g., perceptual circuits in the occipital/temporal lobes or motor circuits in the frontal lobe [3]), or in* specific body representations deficits *(i.e., NA and/or BS deficits), linked to grey matter loss in specific areas underpinning selectively NA (e.g., somatosensory primary cortex and supramarginal gyrus) or BS (e.g., primary motor area and right extrastriate body area) representations [3]. All of these hypotheses need to be verified with studies assessing NA and BS in the same participants and relating the observed performance with the corresponding grey matter alterations. Here we aim to help filling this literature gap by investigating the association between BS/NA performance and grey matter loss in a voxel based morphometry (VBM) analysis study on individuals with lower limb amputation.

## 2. Methods

### 2.1. Participants

Participants included 14 male individuals with lower limb amputation (LLA) and 11 healthy male controls (HC) matched for age (LLA: average: 45.50, SD= 17.90; HC: average= 41.82, SD= 15.87; t_(1,23)_ = .54; p= n.s.) and educational status (LLA: average: 12.43, SD= 3.65; HC: average= 15.00, SD= 3.26; t_(1,23)_ = -1.86; p= n.s.). The LLA sample was also recruited for another study carried out by our research group [21].

All participants showed normal reasoning skills in an abstract reasoning task [25] and none of them had any history of neurological or psychiatric disorders.

In the LLA group, the average time since amputation was 1631 (±2197) days. The amputation was mainly due to trauma (n = 9). The side of amputation was right in 9 and left in 5 LLA and the level of the amputation was transtibial in 6 LLA and transfemoral in 8 LLA. Phantom limb phenomena were present in all LLA. Eight out of 14 LLA had been fitted with prostheses while six of them had never used a prosthesis. For more details about demographics and clinical data on the LLA group see Table 1.

The study was designed in accordance with the principles of the Declaration of Helsinki and approved by the local ethical committee of IRCCS Fondazione Santa Lucia of Rome. All participants gave their written informed consent to participate in the study.

Data are fully available upon request to the corresponding author.

### 2.2. Behavioural Testing

#### 2.2.1. Assessment of Non-action Oriented Body Representations

NA was assessed using the “Frontal body-evocation” subtest (FBE) of the Body Representation Test [18], which evaluates the so-called visuospatial body map or structural body description [10, 11]. This test has been extensively used to assess body representation deficits in adults with brain-damage [5, 6, 10, 26, 27] or limb amputation [10].

**Figure 1:**
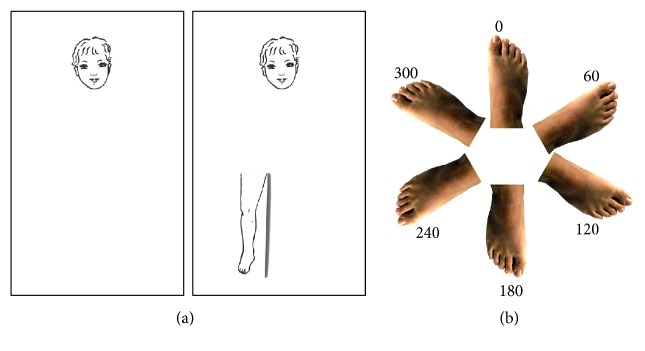
(a) Frontal body-evocation subtest [13] for the assessment of the NA representations. An example of tile positioning is provided on the right of the panel. (b) Items of the Mental Rotation of Feet task for the assessment of the BS representation.

Test materials include a small plastic board on which the position of the head was depicted as a reference, and nine tiles, each representing a body part (Figure 1(a)). Participants were presented with one tile at a time. Their task was to identify the body part depicted on the tile by naming it (i.e., naming score) and then to place it on the board (i.e., localization score). The position of each tile was recorded by the examiner overlapping on the board a transparent sheet on which was depicted a grid that helped the examiner to code the exact position of the tile and to score the task. Then, the examiner removed the tile located by the participant before showing the next tile. The number of correct answers was recorded (naming score: max score= 9; localization score: max score= 9). Naming score was taken as an index of semantic NA, whereas localization score was taken as an index of visuospatial NA.

#### 2.2.2. Assessment of Body Schema Representation

BS was assessed using a mental rotation of feet task (adapted from [17]). Tasks that involve the mental rotation of body parts are considered to assess BS being tasks in which the mentally simulated kinematic configuration of the body matches the actual current kinematic configuration of one's body (for studies in which mental rotation tasks were used to assess BS see for example [7, 11, 14, 28]. In other words, they are tasks that can be solved by imagining performing the actual movement, thus involving the use of the body schema, as for the actual movements [11, 14, 17]. The set of stimuli included 6 pictures of a foot (Figure 1(b)) with different degree of rotation (0, 60, 120, 180, 240, and 300 degree) and laterality (left and right foot). Stimuli were 500 per 500 pixels (width and height) and were presented on a white background in an unbroken sequential manner. Each stimulus was presented 10 times for 3 s, followed by a jittered intertrial interval, ranging from 1.5 to 3 s, during which a fixation point was shown. In each trial participants were asked to decide, as rapidly and accurately as possible, whether the presented stimulus was a right or a left foot, pressing the corresponding button on a keypad. The experiment was implemented in Matlab, using Cogent 2000 (Wellcome Laboratory of Neurobiology, UCL, London, http:// http://www.vislab.ucl.ac.uk/cogent.php). Accuracy of each participant was calculated as the proportion of correct responses. This value was submitted to arcsine transformation and used for the statistical analysis [16].

### 2.3. Neuroimaging Investigation

#### 2.3.1. Image Acquisition

A Siemens Allegra scanner (Siemens Medical Systems, Erlangen, Germany), operating at 3 T, was used to acquire magnetic resonance images. Head movements were minimized with mild restraint and cushioning. We acquired a three-dimensional high-resolution T1-weighted structural image for each LLA participant (Siemens MPRAGE, 176 slices, in-plane resolution 0.5 × 0.5 mm^2^, slice thickness 1 mm, TR 2 s, TE 4.38 ms, flip angle 8 deg).

#### 2.3.2. Voxel Based Morphometry Analysis (VBM)

We performed a VBM analysis on LLA participants' T1-weighted structural images, using the VBM8 Toolbox, implemented in SPM8. The T1 anatomical images were manually checked for scanner artifacts and gross anatomical abnormalities. Images were then normalized using high-dimensional DARTEL normalization, segmented into grey matter (GM), white matter (WM), and cerebrospinal fluid (CSF), and then smoothed (FWHM 8 mm). Due to the absence of significant difference between right and left LLA participants, we mirrored MRI scans of LLA participants with right side amputation in the sagittal plane in order to normalize all LLA participants to one side (see [21, 22] for similar methodology).

We performed a multiple regression analysis on smoothed GM images of LLA, including performances on BS and NA (both naming and localization scores were included as predictors). The resulting statistical parametrical maps were thresholded at the cluster level using a false discovery rate (pFDR<0.05), after forming clusters of adjacent voxels surviving a threshold of p < 0.001 uncorrected [29].

## 3. Results

### 3.1. Behavioural Results

**Figure 2:**
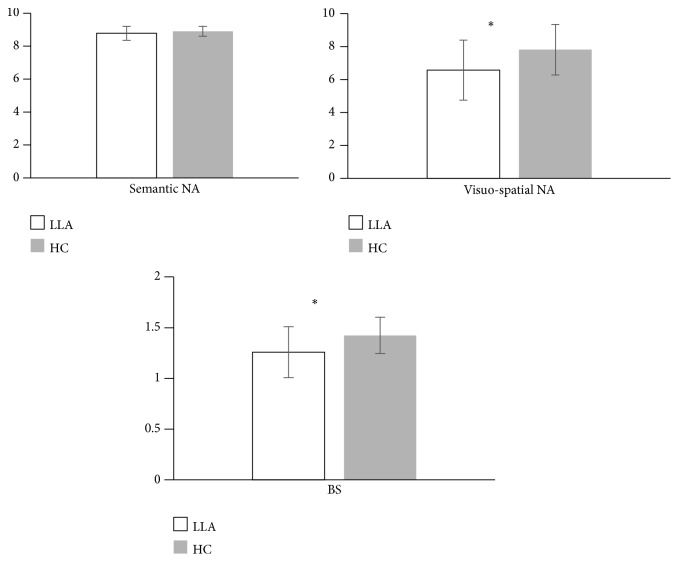
LLA and HC performance on the different body representation tasks.* Notes.* LLA=Lower limb amputees; HC= healthy controls; BS= body schema; NA= non-action oriented body representation.

Based on previous studies from our [10] and other groups [14, 16] finding that amputees performed worse than controls on body representations tasks similar to those used here, we expected that LLA performed worse than C. To test such a hypothesis, we performed one-tailed t-tests on the FBE scores and mental rotation scores. We found significant differences between controls (HC) and LLA (see Figure 2), being LLA performance significantly poorer on both the localization score of the visuospatial NA measure (LLA: 6.57 ± 1.83, HC: 7.82 ± 1.54; t_(1,23)=_ -1.8; p= .04) and on the BS measure (LLA: 1.26 ± .25, HC: 1.42 ± 0.18; t_(1,23)=_ -1.91; p= .03). No statistically significant differences between groups were detected on the measure probing the semantic NA (LLA: 8.79 ± 0.43, HC: 8.91 ± 0.30; t_(1,23)=_ -0.85; p= .20).

Furthermore, Pearson correlation coefficients were computed among the different behavioural measures separately for the HC and LLA in order to verify possible different patterns of correlations. Considering current literature, as expected, in the HC we found a significant correlation between the visuospatial NA and the semantic NA (r= .82; p=.002), while no significant correlations were found between the visuospatial NA and the BS performance (r= .39, p = .23), and the semantic NA and the BS performance (r= .38; p=.24). Interestingly in the LLA group we found a different pattern of significant correlations. Specifically, the visuospatial NA and BS performance were significantly correlated (r= .54, p < .05), but not the visuospatial NA and the semantic NA performance (r= .37; p=.20) or the semantic NA and the BS performance (r= .01; p=.96).

To evaluate the impact of the amputation level on the behavioural performance in the LLA group we performed two tailed t-tests on the two measures in which we found a significant difference between LLA and HC (i.e., the visuospatial NA measure and the BS measure) subdividing the LLA participants in transtibial LLA (N=6) and transfemoral LLA (N=8). No significant differences were found between the two LLA groups (*visuospatial NA measure*: transtibial LLA: 6.50 ± 2.43, transfemoral LLA: 6.63 ± 1.41; t_(1,12)=_ -.12; p=.91;* BS measure*: transtibial LLA: 1.25 ± .19, transfemoral LLA: 1.27 ± 0.30; t_(1,12)= _-.13; p= .90). Similarly, we also performed a t-test to evaluate possible difference between amputees who had lost their dominant/non-dominant limb subdividing the LLA participants in LLA with non-dominant limb amputation (N=9; all had a left amputation) and LLA with dominant limb amputation (N=5; all had a right amputation). Although the dominant LLA showed lower scores on the behavioural tasks, the difference between the two groups was not statistically significant (*visuospatial NA* measure: non-dominant LLA: 7.0 ± 1.73, dominant LLA: 5.80±1.92; t_(1,12)=_ 1.20; p= .26;* BS measure*: non-dominant LLA: 1.31 ± .29, dominant LLA: 1.17 ± 0.15; t_(1,12)= _1.02; p= .33).

**Figure 3:**
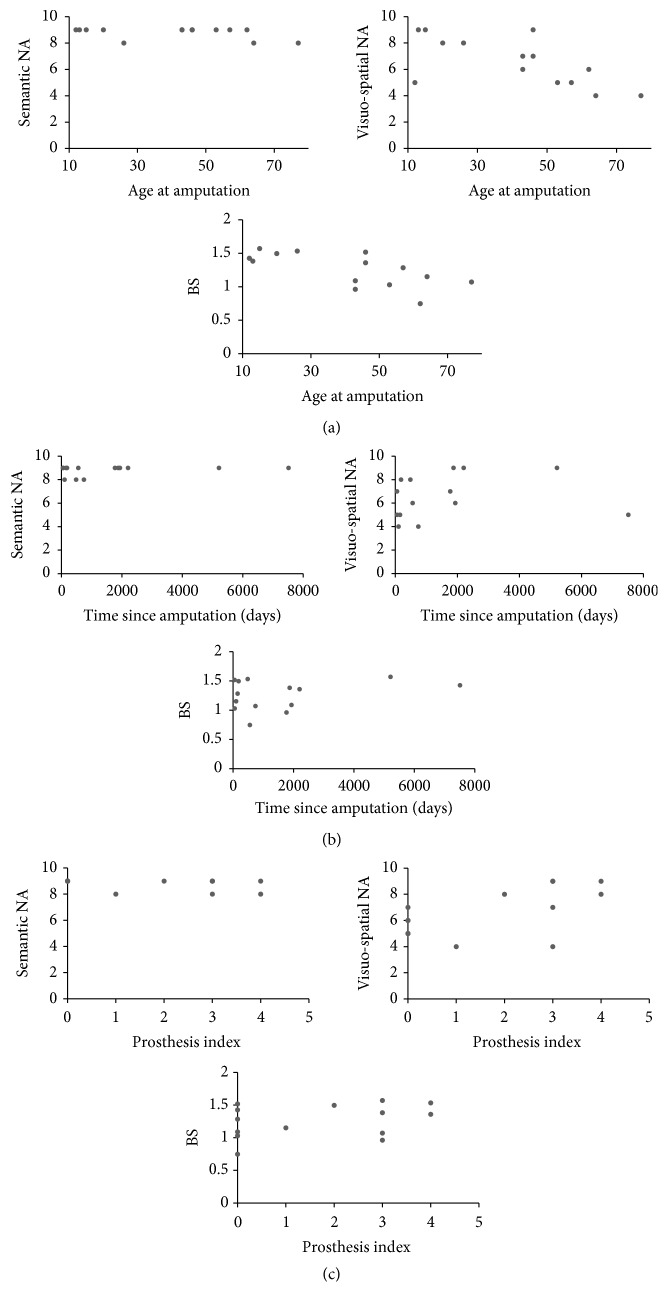
Scatterplots for the relations between age at amputation (panel a), time since amputation (panel b), prosthesis index (panel c), and performance on the different body representation tasks in lower limb amputees.* Notes.* BS= body schema; NA= non-action oriented body representation.

In the LLA group, to evaluate the impact of the time since/age at amputation on the behavioural performance, Pearson correlation coefficients were computed between the scores on the FBE measures and mental rotation task and the time since/age at amputation (see Figure 3). No significant correlations were found between the time since amputation and the BS measure (r = 0.31; p= .28), the semantic NA measure (r = 0.29; p= .31), or the visuospatial NA measure (r = 0.16; p= .57). Similarly, no significant correlations were found between age at the amputation and the semantic NA measure (r = -0.37; p= .19). Instead significant correlations were found between the age at amputation and the BS measure (r = -0.68; p< .01) and the visuospatial NA measure (r = -.68; p< .01).

To evaluate the effect of the amount of the prosthesis use on performance, nonparametric correlations (Spearman's rho correlations; r_s_) were computed between the scores on the FBE measures and the mental rotation task and a prosthesis use index calculated with a procedure inspired by Preißler et al. [23]. In particular, a five points categorical scale was used to classify daily amount of prosthesis use with 0 = never, 1 = 1–3 hours, 2 = several hours but not continuously, 3 = continuously for either the whole morning or the whole afternoon, and 4 = from morning to night. The performance on task tapping on the visuospatial NA significantly correlated with the daily amount of prosthesis use expressed by the prosthesis index (r_s_ = 0.56; p= .04). Instead, no significant correlations were found between the index of prosthesis use and the BS measure (r_s_ = 0.29; p= .31) or the semantic NA measure (r_s_ = -0.39; p= .17). See Figure 3.

### 3.2. Neuroimaging Results

**Figure 4:**
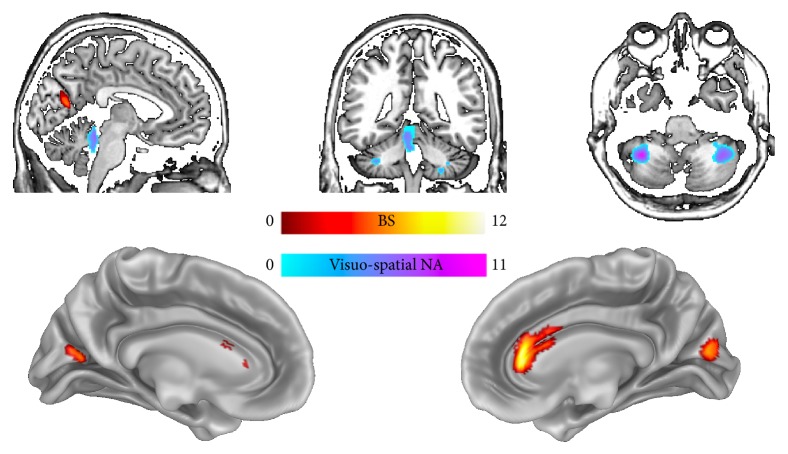
Grey matter volume associated with BS measure (red-to-yellow patched) and visuospatial NA measure (light blue-to-violet patches) in lower limb amputees, as it results from the voxel-wise multiple regression analysis.* Notes.* BS= body schema; NA= non-action oriented body representation.

The multiple regression analysis shows a positive association between GM volume of the vermis (extending to the left lobule IV-V), the left (lobule VIII) and right (crus II) cerebellum, and visuospatial NA measure, as well as a positive association between the GM volume of the right anterior cingulate cortex and the bilateral cuneus (Table 2; Figure 4) and BS measure. No significant association was detected for semantic NA.

## 4. Discussion

We carried out an investigation of body representations, evaluating both the supporting actions (BS) and the non-action oriented (NA) body representations, in a group of individuals with lower limb amputations and assessing the association of BA and NA alterations with grey matter loss. This study sheds more light not only on the cognitive effects of limb loss, but also on our more general and theoretical understanding of body representation.

We have shown behavioural impairments both in BS and visuospatial NA tasks within the same sample of LLA participants. This pattern of results is consistent with those of previous studies, in which however just one type of body representation deficit (BS or NA) was investigated [10, 14–16]. Indeed, in present study the LLA group showed a poor performance in tasks involving mental rotation of body parts (this is consistent with [14–16] and in processing NA representation in terms of visuospatial processing of body part relations (i.e., localization score of the “Frontal Body Evocation” subtest; this is consistent with our previous study on a different LLA sample [10]). We found also a significant correlation between the behavioural performance in BS and visuospatial NA measures which, as expected considering the current neuropsychological and fMRI literature, was not present in healthy controls. The lower performance of LLA as well as the peculiar interplay between BS and visuospatial NA in LLA deserves a feasible explanation. These results would suggest that a body loss can result in a general body representation deficit that affects both BS and visuospatial NA representations, sparing a more semantic component (i.e., no deficits in the semantic NA representation). Theoretically, such behavioural scenario would predict that BS and NA deficits following lower limb amputation are related to a loss of grey matter volume in a brain network that is generally involved in body representations (i.e., in brain areas which underpin both BS and NA; [3]) and partially overlapping. However, the present VBM data show that BS and visuospatial NA performances in LLA predicted a decrease of grey matter volume in brain areas at least in part not overlapping. This result suggests that the loss of a limb affects the two body representations possibly trough different alterations that reflect the deprivation/loss of specific information. Indeed, a limb amputation results in the loss of sensorimotor inputs which are crucial to update/maintain the BS representation, but also in the loss of proprioceptive and visual inputs which are crucial to build up the more visuospatial and structural components of the NA representation (i.e., visuospatial body map or structural body representation). The loss of these different sources of “peripheral” inputs results in a loss of grey matter in specific brain areas devoted to process such different inputs and, in turn, in different behavioural body representation deficits (or,* vice versa*, the loss of different source of “peripheral” information could result in specific behavioural body representation deficits and in turn in a loss of grey matter in different brain areas).

Specifically, concerning* body representation supporting actions*, the multiple regression analysis showed significant association between BS measure and the grey matter volume in the right anterior cingulate cortex (ACC). The involvement of this area, which is crucial in the processing of motor and sensory inputs, is consistent with the fact that the BS is a dynamic representation, built up from multiple sensory and motor inputs, that interacts with the motor system to generate actions [30]. Indeed, the dorsal ACC (also called caudal ACC or middle cingulate cortex, MCC; for details see [31]) has extensive connections with motor-related areas (e.g., premotor and primary motor areas) and with both pain- and motor-related thalamic nuclei and contains the cingulate motor areas, which project to the spinal cord [31]. Interestingly the anterior cingulate cortex together with the insula and other frontal areas is also involved in self-body processing and recognition [32]. We have also showed an association between BS and the caudate nucleus, which contributes to implement the correct action schemas and to select appropriate subgoals based on an evaluation of action-outcomes [33]. This is consistent with fMRI studies on motor imagery [34, 35] and underlines the importance of processing motor information to build up BS.

Concerning the* non-action oriented body representations*, the multiple regression analysis showed that lower visuospatial NA measures were predicted by decreased grey matter volume in the bilateral cerebellum. In particular, we found a relation with the lobule VIII, the vermis, and with crus II that according to a recent meta-analysis is considered a “cognitive region” of the cerebellum [36].

Interestingly, a grey matter volume reduction in lobule VIII, a sensorimotor cerebellar region (for a meta-analysis on the functional topography of the human cerebellum see [36]) that we have previously identified as affected by prosthesis use [21], is predicted by lower visuospatial NA measures. This would suggest an involvement of somatosensorial information in building an NA representation. This result is interesting in light of the debate about the nature of the information necessary to build up not action oriented body representations such as the visuospatial body map/structural body representation that here we have investigated. The visuospatial body map/ structural body representation is hypothesized to be primarily based on vision [11, 37], but some authors suggest that both visuospatial and somatosensorial information are crucial in building up such representations [38]. Our anatomical results ties in well with the second hypothesis, which is also supported by a recent study on healthy individuals which demonstrated the relevance of touch and postural information in generating the visuospatial body map/structural body representation [39].

The areas in present study were related to BS and NA do not completely overlap with the ones previously described in a meta-analysis on fMRI studies [3]. Differences can be related to the investigated sample (i.e., amputees in present study vs. healthy participants in the meta-analysis), as well as to the different neuroimaging (i.e., VBM vs fMRI) or behavioural testing methodologies. Indeed, here we evaluated BS only in terms of motor imagery (mental rotation of body parts) and NA only in terms of visuospatial processing of relation among body parts (i.e., a visuospatial map of body) and semantic knowledge while our meta-analysis included studies using a variety of different tasks. This underlines the importance of evaluating body representations with different methodologies to reach a better understanding of this complex process.

Investigating brain modifications triggered by a “peripheral” alteration, we have also provided peculiar knowledge about the way body representations are maintained/updated. Indeed, it is still a matter of debate whether processing the body configuration depends on our daily interaction with our own body or on visually perceiving other peoples' bodies or whether it is hard-wired in the brain [40–42]. According to the latter two hypotheses, patients with limb amputation should not show deficits in building body representations because they can use an innate body representation or the visual information coming from the bodies of other individuals. Our data suggest that, in order to be built up effectively, BS and NA in terms of visuospatial body map require also an intact daily interaction with our body. Our data also suggest that these representations are both highly “malleable” since they can be modified in individuals that have normally developed them after a peripheral deficit such as the loss of a limb. Further, the lack of significant correlations between the time since amputation and the behavioural performance suggests that a body representation alteration could occur immediately (for similar results see [10, 14]). However we found a significant correlation between BS and visuospatial NA performance and age at amputation—that is, the lower the age at the time of amputation the better the behavioural performance —suggesting that an amputation occurring early in life could results in a better outcome, possibly due to brain plasticity mechanisms, and that there are critical periods in life in which amputation could have a worse effect.

In a previous study [21] we showed decreased grey matter volume in the bilateral cerebellum only in LLA who did not use prostheses, suggesting that the use of the prosthesis prevents grey matter reduction. Here we show an additional positive impact of prosthesis use on behaviour, that is it has an impact on visuospatial NA representation. In other words, the use of a prosthesis could create an advantage in processing the spatial relations among body parts by providing crucial visual information in the building up of NA representation and, thus, preventing deficits in its processing. Although further investigations are needed to disentangle the causative role of prosthesis use on body representations and associated brain modifications, and vice versa, this advantage seems to be linked to grey matter modification in the cerebellar regions that we have previously identified as affected by prosthesis use (lobule VIII and crus II [16]), suggesting a relationship between high-level body representations coded in the cerebellum and prosthesis use in amputees.

On the contrary, the use of a prosthesis does not show an important impact on the behavioural performance in a BS task, probably because it does not compensate for the lack of crucial sensorimotor information that guides actions. It would be interesting to verify if and how different kinds of prostheses, which provide a sensorimotor feedback, can ameliorate BS representation.

In present study we evaluated BS using a task involving the mental rotation only of a body part that was amputated in all our LLA (i.e., the foot). Considering that sensory and motor inputs coming from other body parts involved in actions are spared in our LLA (e.g., hands), one could hypothesize that BS deficits are specific for the BS representation involving the feet. However, we cannot exclude the presence of more general BS deficits (e.g., peculiar deficit in mental rotation of other body parts involved in actions) due to brain reorganization. Indeed, in a recent unpublished fMRI study from our group on non-dominant lower limb amputees, involving the mental rotation of both feet and hands, we have found a pattern of brain activities different from that of healthy controls not only for the mental rotation of the foot, that is, the amputated limb, but also for the mental rotation of the hand. Future studies should better clarify this point investigating, for example, both feet and hands mental rotations in bigger sample including participants with dominant/non-dominant amputation of lower and upper limbs.

## 5. Conclusions

In this study we evaluated a homogeneous group of individuals with amputation (e.g., only male participants with lower limb amputation), providing results that are consistent with previous behavioural studies and that fit well with neuroanatomical data. Although the limited number of participants suggests caution in drawing definitive conclusion, present results can have a clinical and theoretical relevance. Indeed, since there are 30 million people with amputation worldwide [43], knowledge about the cognitive impact of limb loss has the potential to improve the quality of life of several individuals.* Clinically*, our results could provide some new insights and ideas in developing rehabilitation protocols in which specific cognitive trainings of body representation could support the motor rehabilitation.* Theoretically*, they add to previous ones, collected with different methods and in different populations, in suggesting that specific and segregated neural networks underlie two different body representations, one specialized in supporting actions and the other in representing the visuospatial features of the body in absence of any action involvement [3]. Up until now, body representation has been mainly investigated by means of fMRI studies in healthy individuals or by means of neuropsychological and neuroimaging studies in brain damaged patients using both single case and group methodologies. The study of body representations after limb loss has offered us the unique vantage point to verify current cognitive and neural models of body representation by allowing to understand how intact brain responds to rough body alterations.

## Figures and Tables

**Table 1:** Characteristics of the participants with lower limb amputation (LLA).

	**Demographic**			**Amputation**						
***Participants***	***Gender***	***Age***	***Education***	***Side***	***Footedness***	***Level***	***Age at amputation***	***Time since amputation***	***Cause***	***Prosthetic use***
		*(years)*	*(years)*				***(years)***	*(days)*		
LLA1	M	48	8	R	R	Transfemoral	43	1933	Traumatic	**No**
LLA2	M	51	8	R	R	Transtibial	46	2202	Traumatic	Yes
LLA3	M	53	18	R	R	Transtibial	53	55	Vascular	**No**
LLA4	M	57	11	R	R	Transfemoral	57	151	Vascular	**No**
LLA5	M	79	13	R	R	Transtibial	77	742	Vascular	Yes
LLA6	M	18	12	L	R	Transtibial	13	1874	Traumatic	Yes
LLA7	M	21	13	L	R	Transtibial	20	184	Traumatic	Yes
LLA8	M	27	13	L	R	Transfemoral	26	484	Traumatic	Yes
LLA9	M	29	16	L	R	Transfemoral	15	5211	Traumatic	Yes
LLA10	M	33	13	L	R	Transfemoral	12	7511	Traumatic	**No**
LLA11	M	46	13	L	R	Transfemoral	46	53	Traumatic	**No**
LLA12	M	47	13	L	R	Transfemoral	43	1770	Traumatic	Yes
LLA13	M	63	5	L	R	Transfemoral	62	556	Vascular	**No**
LLA14	M	65	18	L	R	Transtibial	64	102	Tumor	Yes

**Table 2:** For each behavioral score the table lists the regions where the behavioral performance predicted the gray matter volume (as it results from the multiple regression analysis), its cluster-level FDR-corrected p-value, the cluster dimension (number of voxels), the peak T, and the MNI coordinates.

**Region/Score**	***cluster p(FDR-corr)***	***cluster (k)***	***peak T***	***x***	***y***	***z***
**Visuo-spatial NA**						
Cerebellum 8	0.002	707	11.400	-37.5	-54	-43.5
			4.561	-30	-55.5	-52.5
Vermis 3/Cerebellum 4-5	0.032	311	7.359	-3	-40.5	-18
			5.195	-4.5	-43.5	-28.5
			4.901	-6	-54	-21
Cerebellum Crus2	0.002	627	7.295	40.5	-54	-43.5
			5.044	34.5	-52.5	-55.5
			4.418	27	-42	-51
**BS**						
Anterior cingulum	0.001	802	12.340	6	34.5	12
			9.111	0	24	22.5
			6.079	0	36	18
Cuneus	0.033	372	7.346	6	-76.5	16.5
			5.802	9	-81	22.5
			5.689	-4.5	-67.5	13.5
**Semantic NA**						
	no suprathreshold cluster					

## Data Availability

The data used to support the findings of this study are available from the corresponding author upon request.
